# Non-invasive multiple cancer screening using trained detection canines and artificial intelligence: a prospective double-blind study

**DOI:** 10.1038/s41598-024-79383-2

**Published:** 2024-11-15

**Authors:** Elizabeth Half, Adelina Ovcharenko, Ronit Shmuel, Sharon Furman-Assaf, Milana Avdalimov, Assaf Rabinowicz, Nadir Arber

**Affiliations:** 1 Gastroenterology Unit, Rambam Health Care Campus, Haifa, Israel; 2 MIDGAM, The Israel Biobank for Research, Rehovot, Israel; 3 Medical consultant (independent), Tel Aviv, Israel; 4 Medical writer and consultant (independent), Tel Aviv, Israel; 5 SpotitEarly Ltd, Kibbutz Hama’apil, Israel; 6 Integrated Cancer Prevention Center, Tel Aviv Souraski Medical Center, Tel Aviv, Israel; 7 Faculty of Medical and Health Sciences, Tel Aviv University, Tel Aviv, Israel

**Keywords:** Artificial intelligence, Detection canines, Cancer screening, Lung, Breast, Colorectal, Prostate, Volatile organic compounds, Early detection, Colorectal cancer, Breast cancer, Cancer screening, Gastrointestinal cancer, Lung cancer, Cancer, Prostate cancer

## Abstract

The specificity and sensitivity of a simple non-invasive multi-cancer screening method in detecting breast, lung, prostate, and colorectal cancer in breath samples were evaluated in a double-blind study. Breath samples of 1386 participants (59.7% males, median age 56.0 years) who underwent screening for cancer using gold-standard screening methods, or a biopsy for a suspected malignancy were collected. The samples were analyzed using a bio-hybrid platform comprising trained detection canines and artificial intelligence tools. According to cancer screening/biopsy results, 1048 (75.6%) were negative for cancer and 338 (24.4%) were positive. Among the 338 positive samples, 261 (77.2%) were positive for one of the four cancer types that the bio-hybrid platform was trained to detect, with an overall sensitivity and specificity of 93.9% (95% confidence interval [CI] 90.3-96.2%) and 94.3% (95% CI 92.7%-95.5%), respectively. The sensitivity of each cancer type was similar; breast: 95.0% (95% CI 87.8-98.0%), lung: 95.0% (95% CI 87.8-98.0%), colorectal: 90.0% (95% CI 74.4-96.5%), prostate: 93.0% (95% CI 84.6-97.0%). The sensitivity of 14 other malignant tumors that the bio-hybrid platform was not trained to detect, but identified, was 81.8% (95% CI 71.8%-88.8%). Early cancer (0–2) detection sensitivity was 94.8% (95% CI 91.0%-97.1%). This bio-hybrid multi-cancer screening platform demonstrated high sensitivity and specificity and enables early-stage cancer detection.

**Supplementary Information:**

The online version contains supplementary material available at 10.1038/s41598-024-79383-2.

## Introduction

Cancer is a leading cause of morbidity and mortality around the world. In 2022, an estimated 20 million new cancer cases were identified and 9.7 million cancer deaths occurred.^1^ It is widely acknowledged that early detection of cancer is essential for increasing the probability of survival, as it enables the prevention of tumor growth and metastasis.^2^ Thus, any modality facilitating early tumor detection is valuable in increasing the chance of survival.^2,3^

Multiple guidelines recommend that individuals at risk undergo screening programs to detect cancer at an early stage including screening for lung, breast, colorectal, and prostate cancer in high-risk individuals. Despite their crucial role in saving lives, many screening modalities have significant drawbacks, as some involve exposure to ionizing radiation (such as mammograms and computed tomography), while others require invasive procedures (like colonoscopy). Additionally, low compliance with screening tests is common due to various factors including limited accessibility in underserved and rural areas, screening-related anxiety, lack of understanding of the screening process, and associated costs. At present, many cancers, such as ovarian cancer and pancreatic cancer, do not have recommended screening tests due to the low prevalence of the cancer type or the lack of sensitive imaging methods or biomarkers, which may lead to under-detection.^4,5^

SpotitEarly Ltd. has developed a simple, non-invasive, and self-administered screening method to detect cancer in exhaled breath samples from humans. This screening technology combines canine detection with artificial intelligence (AI). The technology relies on three principles: the distinct molecular profile of cancer in breath samples, the canines’ odor sensory abilities to detect this molecular profile, and the use of AI for analyzing canines’ input in order to determine the presence of cancer.

The first principle -- the existence of a distinct molecular profile of cancer in breath samples -- is supported by clear evidence suggesting that tumor cells and their microenvironment produce a unique volatile organic compound (VOC) pattern that is further excreted and can be detected.^6^

The second principle -- the canines’ odor sensory abilities to detect the cancerous VOC profile -- has been assessed using different biological samples such sweat,^7^ blood,^8–10^ saliva,^11^ urine,^12–15^ feces,^16^ and breath.^16–18^ Studies have indicated that breath samples often exhibit superior sensitivity and specificity compared to urine.^19–21^ The performance of canine scent detection may be influenced by several factors, including the training methodology and the genetic characteristics of the canines.^18,20^ Despite promising results, there is a noted heterogeneity in performance across studies,^16,19–23^ and the chemical compounds that canines detect are not well understood.^18,24,25^ Furthermore, only a few studies have been conducted in double-blind screening-like situations, which are essential for validating the clinical relevance of canine scent detection for cancer screening.^25,26^

The third principle -- the use of AI for analyzing canines’ input to determine the presence of cancer -- relies on the pivotal role that AI tools play in modern applications for enhancing both automation and accuracy.^27,28^

This paper presents the results of a double-blind study to evaluate SpotitEarly’s method in detecting multiple types of cancer.

## Materials and methods

### Participants and setting

The study (ClinicalTrials.gov ID: NCT06255041) was conducted from December 2021 to December 2023 at three clinical sites in Israel. The study was performed in accordance with the Declaration of Helsinki. The study was approved by the local institutional ethics committees of Tel Aviv Sourasky Medical Center and Rambam Health Care Campus (approval numbers 0604-20-TLV and 0629-22-RMB, respectively). Samples were also obtained at Hadassah Medical Center from the Israel National Biobank for Research (MIDGAM; https://www.midgam.org.il/ ) under ethics committee approval number 0604-20-TLV. These samples were collected from patients who provided informed consent for collection, storage, distribution of samples, and data for use in future research studies. All breath samples in the study, including those for canine training, maintenance of canine detection abilities, and double-blind study, were provided after the participants gave their informed consent before enrolling in the study.

The study population included males and females 18 years of age or older who attended extensive cancer screening at an integrated cancer prevention center (cancer screening arm) or underwent a biopsy for a suspected malignancy at the study sites (enriched arm). 

To prevent any confounders that might impact the VOCs collected in the breath test, participants were excluded from participation in the study if they had smoked less than two hours prior to providing the breath sample, or if they had coffee or an alcoholic beverage, or had eaten a meal less than one hour prior to providing the breath sample. Participants were also excluded if they were diagnosed and treated for cancer in the seven years prior to the study (except for non-metastatic skin tumors that were surgically removed), had chemotherapy in the last 7 years, underwent a medical procedure in the thorax or airways in the two weeks prior to providing the exam, had an ongoing helicobacter pylori infection or a stomach ulcer, had an intestinal bowel disease flare or an ongoing active infection.

## Sample collection and processing

After providing informed consent, participants were asked to wear a surgical mask and to inhale and exhale normally through the mouth for 5 min. The surgical mask was then sealed in two plastic bags and stored at room temperature. The samples were sent to SpotitEarly’s laboratory, registered in the laboratory’s proprietary internal management application and prepared for the test according to the laboratory operating protocols. Sample collection, delivery, and storage were managed according to validated laboratory work instructions designed to preserve sample quality for up to three months after collection.

The results of each participant’s cancer screening tests or biopsy were recorded as negative or positive. Cancer type and stage, according to the American Joint Committee on Cancer (AJCC) cancer staging manual^29^ were also recorded if the sample was positive. Samples of participants identified as having a benign tumor were excluded from the analysis. The results were deidentified and laboratory personnel were blinded to these results and any information that may apply participants’ clinical conditions until their codes were unblinded for analysis. Additionally, a random identification (ID) number was assigned to each sample to ensure that it was not correlated with any clinical confounders.

The sample and data collection processes were managed by the study coordinators and overseen by the clinical monitor to ensure compliance and maintain quality control throughout the study. Additionally, SpotitEarly’s laboratory adheres to the regulatory requirements of the United States Health Insurance Portability and Accountability (HIPAA) Act and the Health Information Technology for Economic and Clinical Health (HITECH) Act. This includes implementing safeguards to prevent unauthorized access to protected health information (PHI), ensuring encryption of PHI, maintaining it in a secure environment, and monitoring access to this secure environment.

## Detection canines

Six Labrador Retrievers were selected according to a canine selection protocol developed by SpotitEarly canine researchers. The canines were bred and grown at the laboratory facility, and were individually housed in large kennels where they received their normal amount of food and water, regular exercise, and optimal care. The housing and husbandry were approved and followed Israeli regulations. The canines were routinely examined by a staff veterinarian and all the canines were healthy. The study conformed to the animal welfare and ethics recommendations of the State of Israel and to the United States Department of Agriculture National Detector Dog Manual. No deprivation or punishment was used.

The canines were trained to mark a sample as positive for cancer through a distinct behavioral cue - sitting beside the sample immediately after sniffing. Marking the sample as negative is done by continuing to the next sample without sitting. This action lasts less than a second. For this study, the canines were trained to detect malignant lung, breast, colorectal, and prostate tumors. All canines were handled by professional canine trainers during canine training and double-blind testing sessions. A total of 147 cancer-positive samples from all four cancer types and 340 cancer-negative samples were used for training the canines to detect these types of cancers over a period of 6 months. To ensure unbiased results, the samples used for training the canines were distinct from those used in the double-blind testing phase of the study. In addition, to maintain the canines’ high performance level, maintenance training sessions were conducted throughout the clinical trial. These sessions utilized samples specifically designated for this purpose and were not included in the double-blind sample set. To ensure optimal performance, training and maintenance were conducted based on protocols developed by SpotitEarly canine researchers.

## Testing room and laboratory monitoring system

**Fig. 1:**
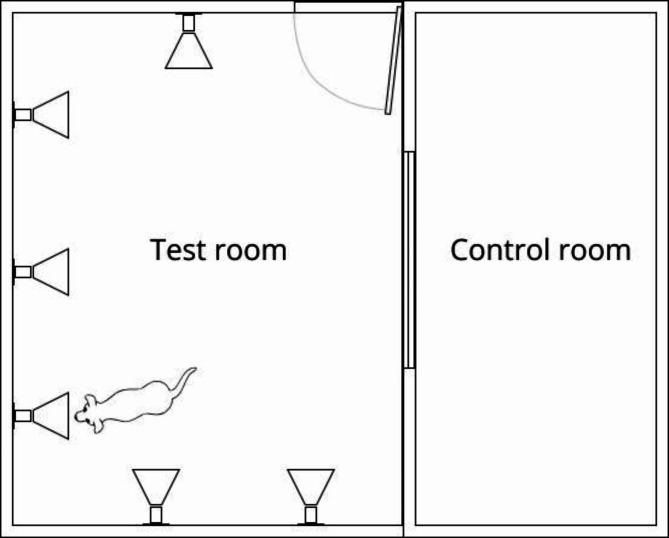
An illustration of SpotitEarly’s testing and control (monitoring) room.

The testing room includes multiple portable sniffing ports, each containing one sample at a time. The room is equipped with sensors and cameras that collect and stream in real-time canine physical and behavioral data to SpotitEarly’s internal application (Fig. 1). This information serves various purposes, such as identifying unusual canine behaviors that may indicate inattention or hesitation. When an unusual behavior is noticed, an alert is sent to the test manager, who reacts accordingly. In addition, the internal application monitors and visualizes the test dynamics in real time, maintaining a record of the tests. This allows the laboratory’s staff to review the tests. The activity in the testing room is monitored from a control room.

### Cancer detection by the bio-hybrid platform

Each detection test comprised six double-blind samples with varying numbers (0–2) of cancer-positive samples. These six samples were evaluated by multiple bio-hybrid units, each consisting of a canine and AI tools. The bio-hybrid units operated independently of one another.

The canines entered the testing room one by one to test all six samples that were loaded to the sniffing ports (Fig. 1). The canine sniffed each sample multiple times, while the AI tools analyzed the canine’s conditioned response (sitting), alongside behavioral and physiological unconditional responses monitored by sensors and cameras in the detection room. This process yielded a cancer prediction status (positive or negative) for each sample.

The bio-hybrid algorithm consists of two main AI components. The first component is an abnormal sniff predictive model, which evaluates each sniff in real-time. This allows the test manager to continuously monitor canine performance and respond promptly as needed, in accordance with the laboratory’s work instructions. The second component is a cancer prediction model, which provides a cancer prediction score for each sample immediately after the test, based on the canine’s behavior and overall body language. Technical details of the modeling framework are described in the Supplementary Data.

Four bio-hybrid units operated sequentially and independently, each with a different canine. The test result for each breath sample was determined based on the majority consensus among the four bio-hybrid units. In the event of a tie — where two units predicted a positive sample and the other two predicted a negative sample — an additional unit was assigned to determine the final sample test result. The test result indicated the presence of cancer but did not specify the cancer type.

## Outcome measures

Each participant’s sample test result (determined by SpotitEarly) was compared to its standard-of-care cancer screening or biopsy result.

The primary outcome of the study was to estimate the test’s sensitivity and specificity for the four types of malignancies that the bio-hybrid platform was trained to detect (breast, lung, colorectal, and prostate). Sensitivity is the number of samples that are correctly detected by the test as positive (true positive) out of all clinically positive samples. Specificity is the number of samples that are correctly detected by the test as negative (true negative) out of all clinically negative samples.

Secondary outcome measures included the sensitivity of each of the four cancer types, and the sensitivity of early-stage detection (stages 0–2; stage 0 was only relevant for breast and lung cancer) -- both collectively and individually for each cancer type.

An exploratory outcome was the ability of the the bio-hybrid unit (i.e. the canine plus AI) to detect other malignancies, in addition to the four it was trained to detect. Another exploratory outcome was the assessment of test repeatability using randomly selected samples that were tested twice using the same or different canines.

### Simulation of a new experimental AI algorithm

In addition to the AI algorithm that was used to generate SpotitEarly’s test results, a new algorithm was developed after study completion using the same modeling framework (Supplementary Material). This algorithm incorporates additional data, such as participant demographics and medical history, which were not available during the study due to blinding. Moreover, it features a new model architecture that diverges from the bio-hybrid majority consensus approach. Instead, it utilizes a distinct modeling layer to aggregate information from the bio-hybrid units in a more sophisticated manner. This aggregation efficiently weights the bio-hybrid units, making the test results less susceptible to the natural variance in canine performance.

The cancer predictive model assigns a score to each sample, which is then compared to a predefined threshold. If the score exceeds the threshold, the sample is predicted as positive; conversely, if the score is equal to or is lower than the threshold, the sample is predicted as negative. The determination of the threshold aligns with product requirements, balancing target sensitivity and specificity. The relationship between the threshold, sensitivity, and specificity is pivotal: an increased threshold value corresponds to decreased sensitivity and increased specificity, and vice versa. This relationship can be visualized by the receiver operating characteristic (ROC) curve. The evaluation of the model is conducted by computing the area under the curve (AUC).

The experimental bio-hybrid algorithm was compared to the current bio-hybrid algorithm that provided the test result, as well as to a simulated simple methodology without using AI tools, where cancer prediction is based solely on the conditional response of a single canine, i.e., the test is positive if the canine sits next to the sample after sniffing it, whereas the test is negative if the canine does not sit next to it after sniffing it.

### Statistical analysis

Test sensitivity and specificity measures were estimated along with their 95% confidence interval (CI) which was calculated using the Wilson score interval.

The repeatability of the test was assessed by calculating the proportion of consistent results of randomly selected samples that were tested twice. It is important to note that the second test results were only used for repeatability estimation and did not influence the assessment of sensitivity and specificity.

## Results

A total of 1386 participants (59.7% males) with a median age of 56 years (range 22–94 years) were included in the analysis. According to the medical centers’ cancer screening/biopsy results, 1048 participants (75.6%) were negative for cancer and 338 (24.4%) were diagnosed with cancer (Table 1). Among the positive samples, 77 samples (5.6%) were positive for cancer types that the canines were not trained to detect. These samples included the following tumors: kidney, lower urinary tract, ovarian, carcinoid, cervical, endometrial, stomach, oropharyngeal space, vulvar, appendix, mesothelioma, thymoma, thyroid, and pancreatic cancer.

**Table 1:** Demographics and cancer screening/biopsy results of the study participants.

Cancer screening/biopsy results	*N* (%)	Median age (range)	Males, *n* (%)	Females, *n* (%)	Current smokers
**Negative**	1048	54 (22–85)	664 (63.4%)	384 (36.6%)	97 (9.2%)
**Positive**	338				
Breast cancer	80	60 (28–92)	1 (1.3%)	79 (98.8%)	6 (7.5%)
Lung cancer	80	70 (47–88)	34 (42.5%)	46 (57.5%)	20 (25.0%)
Colorectal cancer	30	66 (42–92)	20 (66.7%)	10 (33.3%)	9 (30.0%)
Prostate cancer	71	69 (50–85)	71 (100%)	0 (0%)	9 (12.7%)
Other*	77	67 (28–94)	37 (48.0%)	40 (52.0%)	13 (16.9%)

* kidney, lower urinary tract, ovarian, carcinoid, cervical, endometrial, stomach, oropharyngeal space, vulvar, appendix, mesothelioma, thymoma, thyroid, and pancreatic cancer.

### Sensitivity and specificity of detection by the bio-hybrid platform

The overall sensitivity and specificity of detection of the 4 cancer types that the bio-hybrid platform was trained to detect were 93.9% (95% CI 90.3%-96.2%) and 94.3% (95% CI 92.7%-95.5%), respectively. The overall sensitivity of detection of all cancer types (including those that the canines were not trained to detect) was 91.1% (95% CI 87.6%-93.7%). The sensitivity values of each of the four cancer types were similar, ranging from 90.0% (95% CI 74.4%-96.5%) for colorectal cancer to 95.0% (95% CI 87.8%-98.0%) for lung and breast cancers (Table 2). The sensitivity for detection of other malignant tumors that the canines were not trained to detect was 81.8% (95% CI 71.8%-88.8%).

**Table 2:** Sensitivity and specificity of cancer detection by cancer type.

Cancer screening/biopsy results	Frequency	Sensitivity (95% confidence interval)
All cancer types	308/338	91.1% (87.6-93.7%)
All 4 cancer types	245/261	93.9% (90.3-96.2%)
Breast cancer	76/80	95.0% (87.8-98.0%)
Lung cancer	76 /80	95.0% (87.8-98.0%)
Colorectal cancer	27/30	90.0% (74.4-96.5%)
Prostate cancer	66/71	93.0% (84.6-97.0%)
Other*	63/77	81.8% (71.8-88.8%)

* kidney, lower urinary tract, ovarian, carcinoid, cervical, endometrial, stomach, oropharyngeal <i>space</i>, vulvar, appendix, mesothelioma, thymoma, thyroid, and pancreatic cancer.

Sensitivity results per cancer stage are presented in Table 3. All 9 samples with stage 0 (from breast and lung cancer) were detected (95% CI 70.1%-100%). In addition, stage 1 cancer was detected with a sensitivity of 94.9% (95% CI 89.8%-97.5%), and stage 2 cancer was detected with a sensitivity of 94.1% (95% CI 85.8%-97.7%). The overall sensitivity of early-stage cancer detection (stages 0–2) was 94.8% (95% CI 91.0%-97.1%).

**Table 3:** Sensitivity of cancer detection by stage.

Stage	Frequency	Sensitivity (95% confidence interval)
0*	9/9	100.0% (70.1-100.0%)
I	129/136	94.9% (89.8-97.5%)
II	64/68	94.1% (85.8-97.7%)
0-II*	202/213	94.8% (91.0-97.1%)
III	35/39	89.7% (76.4-95.9%)
IV	8/9	88.9% (56.5-98.0%)

*Only breast and lung malignancies were stage 0

The sensitivity of early-stage cancer detection per cancer type is outlined in Table 4. As depicted in the table, the sensitivity values in early cancer stages closely resemble the overall sensitivity values for each respective cancer type. Moreover, despite early-stage positive samples constituting only a subset of the positive samples, substantial sample sizes exist for early-stage cancer detection across all types except for colorectal cancer. These ample sample sizes allow for precise estimation of early-stage sensitivity values with relatively small confidence intervals.

**Table 4:** Sensitivity of early-stage cancer detection by cancer type.

Type	Frequency	Sensitivity (95% confidence interval)
Breast	61/65	93.8% (85.2-97.6%)
Lung	64/66	97.0% (89.6-99.2%)
Colorectal	19/22	86.4% (66,7-95.3%)
Prostate	58/60	96.7% (88.6-99.1%)

Analysis of the sensitivity and specificity of cancer detection of the 4 cancer types that the canines were trained to detect by age group, gender and current smoking status showed similar values among the different categories (Table 5).

**Table 5:** Sensitivity of cancer detection by age, gender, and current smoking status.

Variable	Sensitivity* n/*N* (%)	Specificity* n/*N* (%)
Gender		
Male	116/126 (92.1%)	624/664 (94.0%)
Female	129/135 (95.6%)	364/384 (94.8%)
**Age category**		
≤ 49 years	26/28 (92.9%)	390/412 (94.7%)
50–64 years	68/73 (93.2%)	473/501 (94.4%)
≥ 65 years	151/160 (94.4%)	125/135 (92.6%)
**Smoking status**		
Current smoker	39/44 (88.6%)	90/97 (92.8%)
Non-smoker	206/217 (94.9%)	897/950 (94.4%)

*The analysis included only the four cancer types that the canines were trained to detect.

### Assessment of test repeatability

The samples of 305 participants were tested twice to evaluate repeatability; among them, the samples of 290 participants (95.1%) showed identical results both times (95% CI 92.0%-97.0%).

### Inference of the AI predictive models

The test results derived from the AI algorithm used in the study were compared with the simulation outcomes of two alternative algorithms: a new AI algorithm developed after study completion using the same modeling framework (Supplementary Data), and a simple method where cancer prediction was based only on the conditional response of a canine-only method (without the use of AI tools). As shown in Table 6, performance outcomes decreased dramatically when AI was not used. Moreover, the simulated outcomes from the new AI algorithm surpassed those of the currently used AI algorithm. This outcome was expected, given the utilization of more comprehensive data and a more adaptable model architecture.

**Table 6:** Comparison of performance outcomes by prediction algorithm.

Metric\Method	Canine-only method	Current AI algorithm	New AI algorithm
Sensitivity	64.4%	93.9%	94.8%
Specificity	93.9%	94.3%	96.2%

**Fig. 2:**
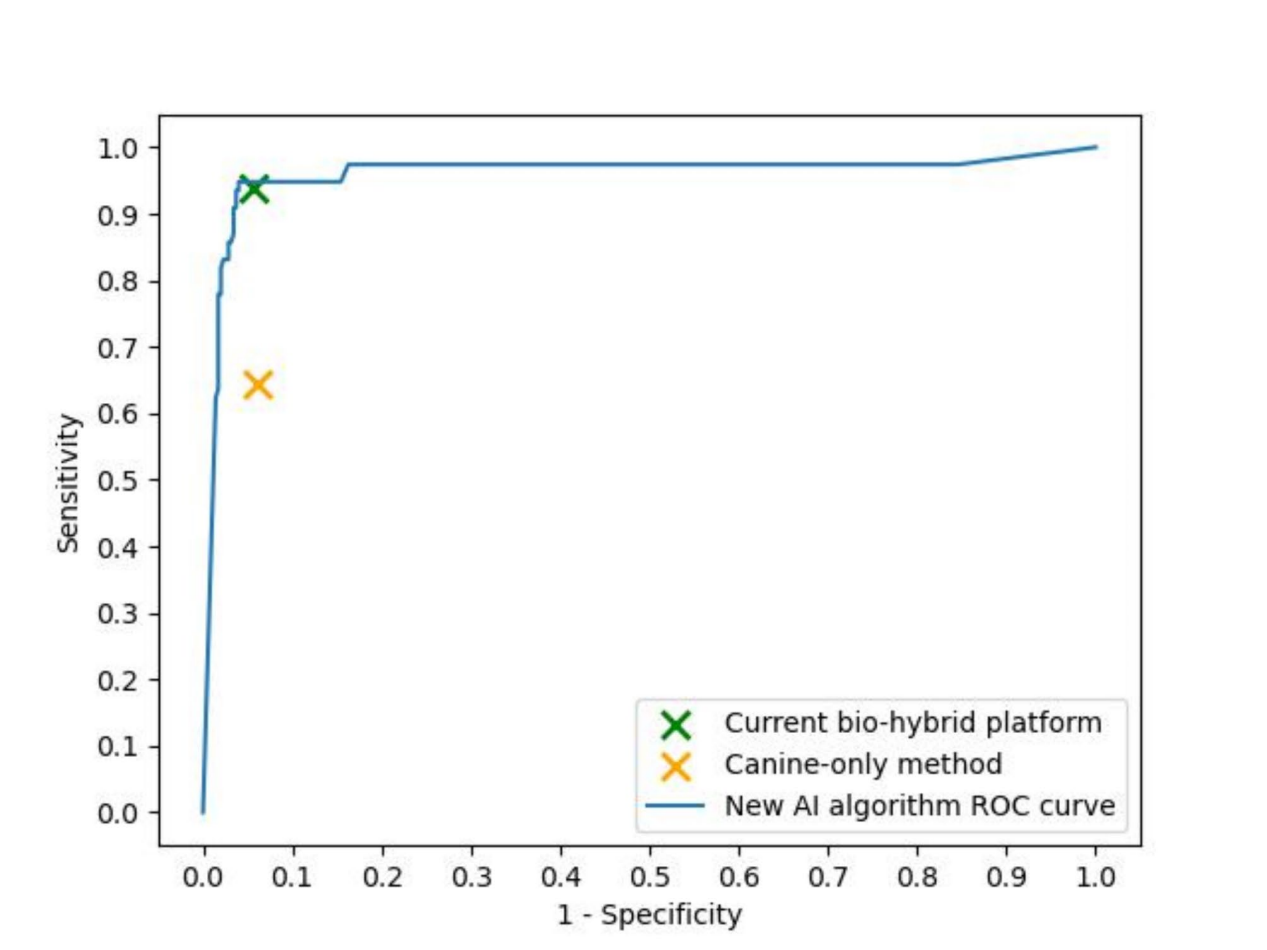
Receiver operating characteristics (ROC) curve of the new AI algorithm (blue line), compared to the test results of the current bio-hybrid model (green X), and canine-only method without AI (orange X).

Figure 2 illustrates the ROC curve of the new bio-hybrid algorithm. The curve’s trajectory demonstrates the tradeoff between sensitivity and specificity resulting from adjusting the model’s threshold, which is selected based on the business strategy. The AUC of the new bio-hybrid algorithm is 98.2%. Both markers for the current bio-hybrid algorithm and the canine-only method lie below the ROC curve and therefore they are inferior in terms of prediction accuracy.

## Discussion

Early cancer detection plays a pivotal role in improving the likelihood of survival by preventing tumor growth and metastasis. The National Cancer Plan released by the National Institutes of Health’s National Cancer Institute in April 2023 has declared early cancer detection as one of its main goals.^30^

The results of the study showed that the SpotitEarly screening test detects malignant lung, breast, colorectal, and prostate tumors in exhaled breath samples with 93.9% sensitivity and 94.3% specificity. Furthermore, the test demonstrates similar performance for early detection of cancer, with 94.8% sensitivity, a characteristic that sets it apart from many other screening tests which often struggle to maintain high sensitivity for early-stage cancer detection without compromising specificity.^31^Maintaining high specificity is crucial for ensuring the test’s reliability. This can be achieved by utilizing the AI part of the bio-hybrid approach, which allows controlling the balance between sensitivity and specificity by adjusting the model’s prediction threshold. Other studies that used canines alone without AI for cancer detection in double-blind settings, reported a sensitivity range of 71–99% and a specificity range of 91–99%.^14,16–18^ However, unlike the current study, they were conducted in non-commercialized settings, where the overall number of tested samples was small and the number of positive samples within a test set was fixed. Other studies, which included varying numbers of cancer-positive samples within a test set - similar to this study, often exhibited limited performance.^26,32^ Furthermore, in the current study the dogs were trained to detect several types of cancer in a single breath sample. The remarkable performance of SpotitEarly’s test in this challenging scenario, surpassing that of previous studies, can be attributed to the innovative bio-hybrid approach. By integrating an AI layer atop the canines for decision-making, data analysis, and real-time monitoring, this approach not only enhances performance but also allows for fine-tuning the prediction algorithm to meet specific product requirements, particularly regarding the sensitivity and specificity optimization tradeoff. Moreover, the AI layer can facilitate the integration of additional data sources beyond canine responses to samples, including patient demographics and medical information. Indeed, our analysis showed that performance outcomes decreased dramatically when AI was not used. Moreover, the simulated outcomes from the new bio-hybrid algorithm, which utilized more comprehensive data and a more adaptable model architecture, surpassed those of the currently used bio-hybrid algorithm. It is important to note that, unlike the AI algorithm employed during the study, the new AI algorithm was developed after the study’s completion and was not tested during the study. Nevertheless, its performance has been evaluated using an unseen test set that simulated a real testing environment.

The sensitivity of SpotitEarly’s tests for the detection of each cancer type was comparable to the sensitivity of gold standard screening tests, namely low-dose computed tomography ^33^ for lung cancer, mammography -- with or without --ultrasonography ^34,35^ for breast cancer, fecal immunochemical tests ^36^ and colonoscopy ^37^ for colorectal cancer, and prostate-specific antigen for prostate cancer.^38^ Hence, it provides a multitype cancer screening test covering all four cancer types in a single test and may potentially demonstrate clinical utility for the test as an adjunct to current screening procedures. As the test is not invasive and the samples can be easily provided even in non-clinical settings, it could potentially increase compliance to cancer screening among the general population by serving as a preliminary screening step to gold standard screening. Furthermore, SpotitEarly’s test yielded high-performance results for 14 cancer types that were not part of the canines’ training. Although the current SpotitEarly test does not support the detection of these 14 cancer types, due to its exploratory nature, this observation suggests that different cancer types may share similar VOC patterns in addition to their specific VOC profile characteristics and therefore can be utilized in the future for expanding SpotitEarly’s cancer screening test to include additional cancer types.

In addition to achieving promising accuracy results, the SpotitEarly test demonstrates notable efficiency that can be utilized for enhancing scaling compared to traditional gold standard screening methods. The rapid decision-making process by canines, occurring in less than a second, enables the testing of a vast number of samples within a short duration. Furthermore, the compact size of the testing environment allows for easy replication, facilitating laboratory scale-up. Other tests for screening of multiple cancers are currently being developed, where the most prevalent test is based on liquid biopsy. Despite their innovative and appealing approach of testing dozens of cancer types in a single blood test, their sensitivity is low, particularly during the early stages when the detection’s impact is most crucial. They fail to detect early cancers due to various limitations, including a low signal-to-noise ratio and the lack of distinct biomarkers for each type of cancer.^39^ The Circulating Cell-Free Genome Atlas study using a multi-cancer detection test Galleri (GRAIL) was a validation study evaluating the role of targeted methylation-based multicancer early detection assay in a healthy population (NCT02889978). The results of the pre-specified sub-study, which included 4077 participants in an independent validation set which included 2823 participants with cancer and 1254 participants without cancer confirmed at one year of follow-up, reported specificity of 99.5% and sensitivity of 51.5% across all cancer types. The sensitivity for stage 1, 2, 3, and 4 cancers was 16.8%, 40.4%, 77.0%, and 90.1%, respectively. The false-positive rate of the test was 0.5%.^40^ The sensitivity of early-stage (I/II) cancer detection in the PATHFINDER study (NCT04241796), which analyzed data of 6621 adults aged ≥ 50 years without signs or symptoms of cancer who underwent testing with the GRAIL multi-cancer early detection test was 12.7%.^41^ The SYMPLIFY study evaluated the GRAIL multi-cancer early detection test in participants with symptoms for potential gynecological, lung, or upper or lower gastrointestinal cancers. Out of 5,461 participants included in the evaluation, 368 were diagnosed with cancer, while a cancer signal was identified in 323 patients, of whom 244 were diagnosed with cancer. The reported sensitivity of the test was 66.3% (95% CI 61.2–71.1), and the specificity was 98.4% (95% CI 98.1–98.8%). Sensitivity increased with increasing age and cancer stage, from 24.2% in stage I to 95.3% in stage IV.^42^ In comparison to these studies, the SpotitEarly test showed improved sensitivity and specificity of cancer detection, specifically at early stages.

The accuracy of the screening test in a fully commercial setting may be different than that obtained in a clinical trial given the exclusion of samples from participants with benign tumors from the enriched arm (biopsy), and participants with active inflammatory disease. The participants in our study were instructed not to eat or drink coffee or alcoholic beverages for one hour before providing the breath sample or to smoke for 2 hours before providing the sample. However, Sonoda et al.^16^ reported that these parameters did not confound canine odor detection of early colorectal cancer. Breath odors associated with dietary factors, such as coffee, garlic, onion or other types of food could also interfere with detection accuracy. However, according to Ehmann et al.,^17^ lung cancer detection was independent of chronic obstructive pulmonary disease and the presence of tobacco smoke and food odors. Similarly, Biehl et al.^43^ found no effect of consumption habits, nutrition, medications or concomitant diseases on canines’ ability to detect lung cancer in breath samples. An additional factor that may affect the accuracy of the test in a fully commercial setting is the potential discrepancy between the study population and the test’s target population, namely,  individuals undergoing cancer screening. The study population consisted of two groups: individuals who underwent screening for cancer, and individuals who underwent biopsy procedures, which were included to increase the number of positive samples in the study (i.e., an enriched arm). The latter group may exhibit characteristics that differ from those of individuals who test positive under cancer screening conditions and comprise the test’s target population. The enrichment of positive samples is a common practice in clinical trials,^44^ implemented to ensure the feasibility of study costs. Such variations in demographic characteristics are expected in clinical trials. For instance, the median age difference between groups is due to the challenge of collecting positive samples from younger individuals who tend to be healthier compared to older individuals. It is important to emphasize that the SpotitEarly test performs consistently across various demographic groups, thereby addressing concerns about potential demographic differences influencing the study’s conclusions.^44^

The current settings were adjusted to the study’s sample collection rate and the overall number of participants. To commercialize the test at scale, several factors are currently undergoing optimization using technological solutions. First, the number of samples tested in the testing room are being increased by connecting each sniffing port to multiple samples (rather than to a single sample, as was done in this study) through an automated monitoring and tracking system that replaces samples within each sniffing port. Such a system enables continuous testing on a larger scale, and therefore reduces the marginal testing time per sample. Notably, fundamental modifications to the test setup and environments necessitate updates to the AI algorithms. Second, sample preparation and streamlining the logistics chain are being enhanced by improving the sample collection kit to reduce the number of steps required for its testing preparation, as well as by implementing automation where feasible. Third, efforts are directed at reducing the number of sniffs and canines required per sample while preserving current performance levels. This could potentially be achieved through advancements in AI algorithms, which may enhance the extraction of valuable information from each sniff, thereby reducing the overall number of sniffs and canines needed.

In conclusion, this study introduces a scalable, low cost multi-cancer screening method of breath samples using a bio-hybrid platform comprising canines and AI that achieves high performance in early-stage detection, within a setup that closely mirrors commercial-phase conditions. These results are expected to pave the way for the development of a new generation of cancer screening tests to enhance cancer screening capabilities.

## Electronic supplementary material

Below is the link to the electronic supplementary material.

Supplementary Material 1

## Data Availability

The datasets generated during and/or analyzed during the current study are not publicly available due to patient confidentiality requirements and the proprietary nature of the data. Further information about the data and conditions for access are available from the corresponding author on reasonable request.
