# Supplementary material for: Non-invasive multiple cancer screening using trained detection canines and artificial intelligence: a prospective double-blind study
Source: Sci Rep. 2024 Nov 15;14:28204. doi: 10.1038/s41598-024-79383-2 (PMC11568277; doi:10.1038/s41598-024-79383-2)
Supplement: Supplementary file 1 — Supplementary Material 1 [file 41598_2024_79383_MOESM1_ESM.docx]

**Supplementary material**

**Technical details of the AI algorithm**

The AI algorithm of Spotitearly’s bio-hybrid platform consists of two main AI components. The first component is an abnormal sniff predictive model, which evaluates each sniff in real-time. This allows the test manager to continuously monitor canine performance and respond promptly as needed, in accordance with the laboratory's work instructions. The second component is a cancer prediction model, which provides a cancer risk score for each sample immediately after the test, based on the canine’s sniffs.

*Data Sources*

The algorithms were trained using a combination of two sample sources: unblinded training samples and double-blind samples. The unblinded training samples were collected both before and during the double-blind phase of the study. The double-blind samples were added to the dataset once they were made available, following two unblinding points - the interim analysis and the end of the study.

*Data Pre-Processing*

The dataset was partitioned into two subsets: a training set (70%) and a test set (30%). The test set, which was unseen during model training, was used to evaluate the performance of the trained model to avoid overestimation of performance due to training overfitting.

The features for the models were developed by SpotitEarly's data science team and canine research team, relying on canine behavioral literature.^1-4^ The features were collected using sensors and cameras deployed in the laboratory, capturing non-conditional behavioral gestures of the canines in response to the sample.

*Model Framework and Evaluation*

The selected model framework is the Gradient Boosting algorithm,^5^ which was implemented using the XGBoost package in Python.^6^ Gradient Boosting is a common choice for tabular data due to its high accuracy, inherent feature selection ability and efficient handling of missing values. For more information about the Gradient Boosting model see.^7^

The training process involved selecting two sets of parameters: hyperparameters, which define the model's structure, and model parameters, such as weights and thresholds. These parameters were chosen sequentially using the training data. First, the optimal hyperparameters were selected from a grid of potential sets of hyperparameter values by a grid-search cross-validation technique.^8^ Once the optimal hyperparameters were selected, the model was retrained on the entire training dataset using the selected hyperparameters. After training, the model’s performance was assessed using the test data, which was not used in the training process and therefore could not enhance performance overestimation due to training overfitting.
It is important to note that, although the target variable classes in the data are imbalanced, the proportion of positive samples is sufficiently large that it does not substantially impact model performance. Consequently, class imbalance correction techniques were not applied.

**References**

.1 Siniscalchi, M., Lusito, R., Vallortigara, G. & Quaranta, A. Seeing left- or right-asymmetric tail wagging produces different emotional responses in dogs. *Curr Biol* **23**, 2279-2282 (2013).

.2 Hasegawa, M., Ohtani, N. & Ohta, M. Dogs' Body Language Relevant to Learning Achievement. *Animals (Basel)* **4**, 45-58 (2014).

.3 Siniscalchi, M., d'Ingeo, S., Minunno, M. & Quaranta, A. Communication in Dogs. *Animals (Basel)* **8**(2018).

.4 Ferres, K., Schloesser, T. & Gloor, P.A. Predicting Dog Emotions Based on Posture Analysis Using DeepLabCut. *Future Internet* **14**, 97 (2022).

.5 Friedman, J.H. Greedy Function Approximation: A Gradient Boosting Machine. *The Annals of Statistics* **29**, 1189-1232 (2001).

.6 Chen, T. & Guestrin, C. XGBoost: A Scalable Tree Boosting System. in *KDD '16: Proceedings of the 22nd ACM SIGKDD International Conference on Knowledge Discovery and Data Mining* 785-794 (2016).

.7 Hastie, T., Tibshirani, R. & Friedman, J. *The Elements of Statistical Learning: Data Mining, Inference, and Prediction, Second Edition*, (Springer, New York, NY, 2009).

.8 Shekar, B.H. & Dagnew, G. Grid Search-Based Hyperparameter Tuning and Classification of Microarray Cancer Data. in *2019 Second International Conference on Advanced Computational and Communication Paradigms (ICACCP)* 1-8 (2019).
